# Cultural beliefs about breast cancer in Vietnamese women

**DOI:** 10.1186/s12905-019-0777-3

**Published:** 2019-06-11

**Authors:** Jong Gun Kim, Hye Chong Hong, Hyeonkyeong Lee, Carol Estwing Ferrans, Eun-Mi Kim

**Affiliations:** 1 Department of Nursing, Hoseo University, 20, 79 Street, Hoseo-ro, Baebang-eup, Asan, Chungcheongnam-do 31499 South Korea; 2 Red Cross College of Nursing, Chung-Ang University, 84 Heukseok-ro, Dongjak-ku, room 401-3, building 106, Seoul, 06974 South Korea; 3 College of Nursing, Mo-Im Kim Nursing Research Institute, Yonsei University, 50 Yonsei-ro, Seodaemun-gu, Seoul, 03722 South Korea; 4 College of Nursing, University of Illinois at Chicago, 845 S. Damen Ave., M/C 802 room 606, Chicago, IL 60612 USA; 5 Department of Nursing, Sunlin University, 30 Chogok-gil, 36beon-gil, Heunghae-eup, Buk-gu, Pohang, Gyeongsangbuk-do 37560 South Korea

**Keywords:** Breast cancer, Cultural beliefs, Health behavior, Public health, Nursing education, Vietnamese women.

## Abstract

**Background:**

This study examined factors influencing cultural beliefs associated with later-stage detection of breast cancer and determined what factors influence those cultural beliefs in Vietnamese women residing in a rural Vietnamese community.

**Methods:**

A cross-sectional survey was conducted with 289 women aged 20–64 years from 12 villages using a self-administered structured questionnaire. Cultural beliefs were measured with a 13-item cultural beliefs scale consisting of four domains—characteristics of breast lumps, self-help techniques, faith-based beliefs, and futility of treatment. Data were collected in February 2017 and analyzed using chi-square tests, nonparametric tests, Fisher’s exact tests, and multiple linear regression analyses with SPSS/WIN 24.0 statistical software.

**Results:**

Although the total score was relatively low (3.4 out of 13), cultural beliefs that could contribute to later-stage breast cancer were identified. Younger women (β = .15, *p* = .016) and women with a lower income (β = .21, *p* < .001) held more erroneous cultural beliefs as compared to their counterparts. Most women believed they would not get breast cancer if they took care of themselves. More than one-third held cultural beliefs about breast lumps, thinking they would need to be painful and/or actively growing to be breast cancer.

**Conclusions:**

The results support the urgent need for education concerning breast cancer health promotion, including breast cancer assessment as well as guidance on evidence-based and up-to-date detection measures to change rural Vietnamese women’s cultural beliefs.

**Electronic supplementary material:**

The online version of this article (10.1186/s12905-019-0777-3) contains supplementary material, which is available to authorized users.

## Background

Breast cancer is the most frequently occurring cancer in women, leading to the highest number of cancer-related deaths globally. Breast cancer comprised about 2.4 million of all cancer cases in 2015 [1], which was a 43% increase from 2005. The incidence of breast cancer is higher in developed countries; however, this increasing trend appears in most regions of the world, including Vietnam [1].

Although the breast cancer incidence in Vietnamese women is lower compared to other Asian countries, the incidence has increased owing to decreased fertility rates and increased obesity in Vietnamese populations [2]. The National Cancer Control Program was introduced in selected regions of Vietnam to reduce mortality and morbidity and to improve the quality of life of patients with cancer [3], and it has established cancer registries and initiated an organized screening program designed to detect breast cancer [3]. However, breast cancer screening (BCS) programs have mostly been implemented with limited populations such as women who reside in urban areas, who can afford the cost of screening because of the lack of a systematic, nation-wide, screening policy [4].

As reported in earlier studies, cultural factors such as conceptualizations of health, illness, beliefs, and values can influence women’s BCS practices in certain populations [5–7]. Among women living in Vietnam, the level of BCS is reported to be relatively low. In a national screening program conducted in 2008–2010, less than 10% of women aged 30–60 years from 10 provinces of Vietnam had a chance to take clinical breast examinations [8]. Only a small proportion of Vietnamese women in transnational marriages living in Taiwan had performed a breast self-examination (BSE) during the past year (25.6%) ―a figure which is even lower on a monthly basis (7.6%) ―and 6.8% of this population underwent mammography during the past 2 years [9]. These low levels of BCS lead to breast cancer symptoms going undetected, contributing to late-stage diagnosis of breast cancer and subsequent poorer outcomes and death [10]. Although a low awareness and lack of knowledge concerning BCS appear to be a key barrier to BCS in women residing in low- and middle-income countries [11], there is still a lack of understanding of the perceptions and attitudes toward BCS among women living in rural Vietnamese areas.

Like women in many other Asian nations, Vietnamese women are in a traditional society that more closely follows ancestral conceptualizations of gender roles. Studies have reported that older Asian women hold more traditional health beliefs, such as fatalism, which influence their perceptions of health, illness, and the use of preventive healthcare methods [12]. Rather than practicing preventative techniques, Asian women tend to believe that events such as falling ill are pre-determined [13]. For Vietnamese women living in the United States, gender roles (i.e., femininity) are associated with a higher probability of having a clinical breast exam, but only for those women who were highly acculturated to the U.S. [14]. However, that same study also found that Vietnamese women in the U.S. with higher collectivist values showed higher efficacy for BCS because these women were motivated to perform BCS and maintain their health owing to their key family roles [14]. These findings demonstrate the importance of culture in BCS practices among Vietnamese women.

Culture is defined as a multidimensional set of shared and socially transmitted ideas about the world, which are passed down from generation to generation [15, 16]. Cultural beliefs are considered a determinant for perceptions of health risks and the practice of health-promoting behaviors in diverse populations [17]. Cultural beliefs related to breast cancer are a critical factor in women’s decisions concerning BCS practices in traditional societies. In a systemic review of 23 studies associated with the influence of cultural factors, the experiences of South Asian women with breast cancer were culturally linked to cancer-related stigma, familial experiences, reliance on religion, and low prioritization of women’s issues in comparison to Western women [12]. Further, previous studies showed that cultural beliefs differ by socio-demographic factors such as age, education level, and healthcare insurance [18].

A recent scoping review of breast cancer in Vietnam suggests that patients’ perceptions of social and cultural aspects related to the later detection of breast cancer should be explored to design appropriate interventions [4]. To our knowledge, however, no studies have specifically addressed the cultural beliefs about breast cancer among women residing in Vietnam. Therefore, we investigated Vietnamese women’s cultural beliefs concerning breast cancer and determined the influential factors per socio-demographic characteristics in a rural Vietnamese community.

## Methods

### Design and sample

A community-based, cross-sectional survey was conducted in a district of the central coastal region of Vietnam, Cam Thuy, which has a population of 3500. Cam Thuy is a rural, impoverished community in Quang Tri Province. The survey targeted women aged 20 to 64 years across 12 villages. This study is part of a group of research projects that examine BCS practices and their determinants. Three-hundred twenty-six women voluntarily agreed to participate in the study. A detailed sample calculation was discussed in the primary research that examined the BCS practices and their determinants [19].

### Instruments

Cultural beliefs were measured with the cultural beliefs scale developed by Ferrans [20, 21]. The scale was designed to measure the extent that cultural beliefs act as barriers to early detection, and thus contribute to a diagnosis of later-stage breast cancer for African American, Hispanic, and Caucasian women. The scale comprises 17 items across four content areas: breast lumps (*n* = 4), self-help techniques (n = 4), faith-based beliefs (n = 4), and futility of treatment (*n* = 5). Each item was measured by a dichotomous response (true or false). Four items related to mammogram were excluded as mammogram service is not available in this study setting. Thus, the total score ranges from 0 to 13, with higher scores indicating that participants hold a greater number of cultural beliefs, while lower scores indicate that respondents hold fewer cultural beliefs.

The cultural beliefs scale was translated using a committee approach translation [22]. Three Vietnamese bilingual experts individually translated the questionnaire and reviewed the Vietnamese version of the questionnaire to modify the translation during the design meeting. These three experts were professors in universities in Vietnam who were familiar with Vietnamese culture and proficient in English. After these processes, some modifications were made by the principal investigators and another local expert in response to feedback from participants through cognitive interviews with five Vietnamese women. The copy of the Vietnamese cultural beliefs scale is available as a Additional file 1

### Data collection

Data were collected in collaboration with the local Women’s Council. The study purpose and survey methods were explained to the president of the Women’s Union, and they agreed to assist with data collection. Subsequently, the female leaders from the 12 villages were recruited from the local Women’s Union and trained by local experts to collect the data. Training included explanations of the study objectives and design, how to describe the questionnaire to participants, and how to obtain consent.

Data collectors visited individuals’ homes and common community areas, such as markets, local events, and member meetings, and distributed questionnaires after explaining the purpose of the study. The survey was self-administered. After collecting participants’ responses in private envelopes, a small gift was provided to all participants. Data collection was performed from February 8–13, 2017.

### Data analysis

Demographic characteristics were calculated using descriptive statistics. Differences in cultural beliefs by age group were tested using chi-square tests and Fisher’s exact tests. Age, income, education, marital status, having medical insurance, menopause status, and history of a breast disease diagnosis did not satisfy the assumption of normal distribution (*p* < .05) when Shapiro-Wilk’s regularity test was conducted; therefore, the Mann-Whitney U test and Kruskal-Wallis test were conducted with Tukey’s post-hoc test post analysis. The cut-off for the monthly average income was based on the report from the Vietnam organization in 2017 [23]. In addition, multiple linear regression analysis was conducted to determine the factors affecting cultural beliefs. The assumption of multicollinearity was met because the variance inflation factor values of all variables were less than 10 (1.04–1.77), and the values of tolerance were greater than 0.1 (0.56–0.95). Statistical software SPSS ver. Twenty four for Windows was used for statistical analyses, with the level of significance set at 5%.

## Results

### Participants’ general characteristics

We carefully reviewed each questionnaire received and excluded 37 participants with insufficient or invalid answers; thus, the data from 289 women were included in the final analysis. As shown in Table 1, more than half of the participants (57.8%) were between 20 and 39 years of age. Notably, nearly 80% of the participants had graduated from secondary school which was s lower than 2010–2011 average (94.3%) of Quang Tri in general [24], whereas 4.8% of the participants had no formal education. The majority of subjects were married (90.7%), had medical insurance (85.8%), were not undergoing menopause (84.1%), and had no history of breast disease (94.1%).

**Table 1:** Differences in Cultural Beliefs by General Characteristics (*N* = 289)

Characteristic	*n* (%)	Cultural beliefs	
		M ± SD	U or χ^2^ (*p* values)
Age (years)			
Young: 20–39	167 (57.8)	3.62 ± 1.68	8169.5
Old: 40–64	122 (42.2)	3.07 ± 1.64	(.004)
Income level			
< AMW	201 (69.6)	3.62 ± 1.60	6605.0
≥ AMW	88 (30.4)	2.86 ± 1.74	(< .001)
Education level			
≥ Secondary	224 (77.6)	3.39 ± 1.68	15.84
Primary school	51 (17.6)	2.86 ± 1.38	
			(.027)
No education	14 (4.8)	4.00 ± 1.70	
Marital status			
Married	262 (90.7)	3.35 ± 1.67	3031.5
Not married	27 (9.3)	3.77 ± 1.80	(.215)
Having medical insurance			
Yes	248 (85.8)	3.39 ± 1.75	5035.5
No	41 (14.2)	3.39 ± 1.20	(.921)
Menopause status			
Yes	46 (15.9)	3.39 ± 1.67	5473.5
No	243 (84.1)	3.39 ± 1.69	(.822)
Diagnosed with a breast disease			
Yes	17 (5.9)	2.82 ± 1.18	1820.5
No	272 (94.1)	3.42 ± 1.70	(.136)

### Differences in cultural beliefs by general characteristics

Participants’ self-reported cultural beliefs significantly differed by age, income, and education (*p* < 0.05) (Table 1). Specifically, the average cultural belief scores of participants under 40 years were significantly higher than the cultural belief scores of participants over 40 years (U = 8169.5, *p* = .004). Level of education were significantly related to cultural beliefs (X^2^ = 15.84, *p* = .027), but there was no difference between groups. Women who earned below average monthly wages (< 2,650,000 Vietnam dollar; VND) [23] had higher cultural belief scores than those with higher income levels (U = 6605.0, *p* < .001).

AMW. average monthly wage (2,650,000 VND = 116.25 USD), M = mean; SD = standard deviation.

### Levels of cultural beliefs

As shown in Table 2, participants’ mean cultural beliefs scores were low (3.4 out of 13). By item, the percentage of responses of “true” ranged from 1.4 to 91.3%. The most agreed item was “If breast cancer is treated correctly, it can be cured” (91.3%) in domain IV (futility of treatment). About 63% of women agreed on the item “If you take good care of yourself, you won’t get breast cancer,” in domain II (self-help techniques). No one gave a response of “true” to an item stating that, “If a woman has enough faith in God, she won’t need treatment for breast cancer” in domain III (faith-based beliefs), and only a few women gave responses of “true” to the other two items in domain III.

**Table 2:** Participants’ Levels of Cultural Beliefs (*N* = 289)

Domain	Item	Response n (%)	
		True	False
I. Breast lump characteristics	1. If a breast lump is not painful, it is not cancer.	100 (34.6)	189 (65.4)
	2. If a breast lump does not get bigger, it is not cancer.	109 (37.7)	180 (62.3)
	3. If a breast lump is touched/pressed often, it will turn out to be breast cancer.	26 (9.0)	263 (91.1)
II. Self-help techniques	5. The more you worry about breast cancer, the more likely you will get it.	23 (8.0)	266 (92.0)
	6. If you take good care of yourself, you won’t get breast cancer.	181 (62.6)	108 (37.4)
	13. If you have a breast lump, a “natural” remedy can help to get rid of it.	42 (14.5)	247 (85.5)
III. Faith-based beliefs	7. Faith in God can protect you from breast cancer.	15 (5.2)	274 (94.8)
	10. If you pray enough, sometimes breast lumps will disappear.	4 (1.4)	285 (98.6)
	14. If a woman has enough faith in God, she won’t need treatment for breast cancer.	–	289 (100.0)
IV. Futility of treatment	11. If breast cancer is cut open in surgery, it will grow faster.	18 (6.2)	271 (93.8)
	15. If a woman is poor, she will not get cured from cancer because she won’t get the best treatment.	108 (37.4)	181 (62.6)
	*16. If breast cancer is treated correctly, it can be cured.	264 (91.3)	25 (8.7)
	17. It doesn’t really matter if you get treated for breast cancer, because if you get cancer, it will kill you sooner or later.	90 (31.1)	199 (68.9)
Total mean score		3.4	–

*Reverse coded

Table 3 shows differing responses between younger (aged 20–39 years) and older (40–64 years) participants concerning cultural beliefs regarding breast cancer. Cultural beliefs involving several items across three domains showed significant age-group differences. Younger women showed a significantly lower level of cultural belief for, “If a breast lump does not get bigger, it is not cancer” than did older women (X^2^ = 11.85, *p* = .001). Moreover, significantly more women in the younger group believed, “If you take good care of yourself, you won’t get breast cancer” (X^2^ = 7.88, *p* = .005) and, “If a woman is poor, she won’t get cured from cancer, because she won’t get the best treatment” to be true as compared to women in the older group (X^2^ = 5.57, *p* = .018). The item, “If you have a breast lump, a natural remedy can help to get rid of it” was perceived as “true” with a marginally higher percentage in the younger women in comparison to the older women (X^2^ = 3.75, *p* = .053).

**Table 3:** Differences in Cultural Beliefs by Age Group (*N* = 289)

Domain	Item	Younger n (%)		Older n (%)		χ^2^ (p values)
		True	False	True	False	
I. Breast lump characteristics	1	59 (35.3)	108 (64.7)	41 (33.6)	81 (66.4)	.09 (.761)
	2	77 (46.1)	90 (53.9)	32 (26.2)	90 (73.8)	11.85 (.001)
	3	11 (6.6)	156 (93.4)	15 (12.3)	107 (87.7)	3.53 (.060)
II. Self-help techniques	5	12 (7.2)	155 (92.8)	11 (9.0)	111 (91.0)	.32 (.570)
	6	116 (69.5)	51 (30.5)	65 (53.3)	57 (46.7)	7.88 (.005)
	13	30 (18.0)	137 (82.0)	12 (9.8)	110 (90.2)	3.75 (.053)
III. Faith-based beliefs	7	10 (5.8)	161 (94.2)	5 (4.0)	120 (96.0)	- (.595^a^)
	10	2 (1.2)	165 (98.8)	2 (1.6)	120 (98.4)	- (1.000^a^)
	14	–	167 (100.0)	–	122 (100.0)	–
IV. Futility of treatment	11	7 (4.2)	160 (95.8)	11 (9.0)	111 (91.0)	2.81 (.094)
	15	72 (43.1)	95 (56.9)	36 (29.5)	86 (70.5)	5.57 (.018)
	16	157 (94.0)	10 (6.0)	107 (87.7)	15 (12.3)	3.54 (.060)
	17	52 (31.1)	115 (68.9)	38 (31.1)	84 (68.9)	.00 (.999)

^a^Fisher’s exact test

The multiple linear regression analysis (Table 4) revealed that age and income level significantly affected women’s cultural beliefs related to breast cancer. Women with a lower level of cultural beliefs were younger (< 40 years) (β = .15, *p* = .016) and with a lower income (β = .21, *p* < .001) in comparison to the reference group when other variables were controlled for. These factors explained 8.3% of cultural beliefs in the context of breast cancer reported by Vietnamese women.

**Table 4:** Multiple Linear Regression Analysis for Factors Associated with Cultural Beliefs (*N =* 289)

Variable	βeta	*P* values
Younger age group (ref: older age group)	.15	.016
Lower AMW (ref: AMW or higher)	.21	< .001
Primary school (ref: no education)	−.04	.562
Secondary school or higher (ref: no education)	.07	.314
Having medical insurance (ref: yes)	−.04	.492

F = 5.13, R^2^ = .083, *p* < .001

Ref = reference group; *AMW* average monthly wage

## Discussion

To improve BCS among Vietnamese women, it is imperative to explore the factors affecting their cultural beliefs in the context of breast cancer. This study evaluated the relationships among socio-demographic variables and cultural beliefs for BCS behaviors in Vietnamese women residing in rural Vietnam. This study is valuable in that it is the first to measure certain cultural beliefs of Vietnamese women, which can problematically contribute to late detection of breast cancer.

Contrary to expectations, we found that women’s total cultural beliefs scores were low on average (3.4 out of 13) among Vietnamese women living in a rural areas. However, a considerable number of participants indicated that they believed several erroneous statements regarding breast lumps, self-help techniques, and futility of treatment for breast cancer. According to culturally sensitive mid-range theories, the strength of cultural beliefs, identity, and values that mediate risk perceptions—such as risk attention and risk awareness—influence health-promoting behaviors [25]. Moreover, previous theories have clearly shown the influence of culture on health behaviors insofar as cultural beliefs triggering or prohibiting certain self-care behaviors in individuals with chronic illnesses [17, 26]. As proposed in a theory based on the cultural determinants of help seeking [27], culturally held beliefs about health problems influence help-seeking behaviors in various sample populations, which has significant consequences on disease incidence worldwide. Thus, the current findings are useful for designing culturally appropriate interventions for this study population.

Regarding differences in cultural beliefs between younger and older women, however, this study showed that younger women had lower levels of cultural beliefs about breast cancer than did older women, including the characteristics of breast lumps. Previous studies have shown that cultural beliefs about breast lumps are much more common in women who are typically defined as disadvantaged population with lower socioeconomic status and less access to healthcare services [28]. Unlike women in Western countries who are diagnosed with breast cancer at a relatively early stage, many cases of breast cancer in Vietnamese women aged 45–55 years are found at a later stage [29]. Thus, women aged younger than 40 years are addressed as a priority population for education on the importance of BCS and the encouragement of BCS adherence. However, since the target women in this study resided in an underdeveloped community with limited health resources, even younger women might have lower awareness of BCS for the early detection of breast cancer. Consistently, Liu and colleagues [30] also showed that about three-quarters of young women aged 25–44 years who lived in rural China, where medical access was limited and health screening was not universal, had poor awareness levels of BCS.

It is noteworthy that younger women were twice as likely as older women to agree with the statement, “If a breast lump does not get bigger, it is not cancer.” As suggested by Lin and colleagues [31], this might be partly due to a lack of information from healthcare providers, messaging that is difficult to understand, or misinterpreted experiences. These cultural beliefs may result in a fear of breast cancer diagnosis that inhibits BCS. In fact, women in Asian societies often express fears that breast cancer diagnosis would disgrace their families, emphasizing the importance of maintaining their social standing within the family [13, 32]. Such fear and stigma around breast cancer diagnosis are possible in Vietnamese communities and thus require further research attention. Further, although advice on BSE remains controversial, in environments with limited resources where women lack opportunities for clinical examination, BSE is the best alternative for the early detection of breast cancer. Increased focus on opting for BSE based on the four-step ladder motivation model (i.e., taking opportunities, clarifying confusion, maintaining health, and illness monitoring) is suggested for BSE interventions in young Vietnamese populations to increase risk awareness and to reduce cultural beliefs regarding breast cancer [33].

In this study, more than half of all the participants, including both younger (69.5%) and older (53.3%) women, believed that they would not get breast cancer if they took good care of themselves. As reported in a recent study of Asian and African women living in Australia [34], this cultural factor plays a key role in shaping breast cancer beliefs and screening behaviors in women. For example, certain groups of women do not realize that they are at risk of getting the disease even though they feel healthy. This finding is similar to a study by Shang, Beaver, and Campbell [32], in which Chinese women recognized the importance of self-care to promote health and prevent illness; however, they did not pay much attention to BSE. Importantly, even in the younger age group in the current study, perceptions regarding self-help techniques were considerably impacted by cultural beliefs, indicating that young women still need to be educated to increase BCS awareness. Using a variety of channels, such as healthcare providers and the media, the development of culturally appropriate health education to increase BCS awareness and to reduce cultural beliefs regarding breast cancer is recommended.

Prior studies [35–37] including community-based population surveys in low- and middle-income countries showed consistent results—that participants’ BCS awareness was significantly lower than it was in developed countries. In a recent review of 53 studies conducted in resource-limited countries [11], lack of knowledge and low awareness of BCS were key barriers to women obtaining cancer screening. This may be a possible consequence of their lower living standards and less supportive public health service. Therefore, it is necessary to provide more effective strategies to promote health service access and health screening to vulnerable and underserved populations who have erroneous cultural beliefs concerning BCS.

The current findings have several practical and research implications. The extent of the breast cancer cultural beliefs identified in this study would be informative to healthcare providers working in community health centers in Vietnam. Healthcare providers should competently provide community education concerning the early detection of breast cancer and diagnostic techniques for BCS practices, which are lacking in Vietnam [4]. This indicates the need for a training program for healthcare providers working in community health centers. Healthcare providers also need to understand the factors influencing women’s cultural beliefs such as age and income level when establishing educational programs. In addition, BCS should be promoted early in clinical settings since Vietnamese women are more likely to be diagnosed with later-stage breast cancer than are Western women. This may require easier access to clinical settings in rural areas at a low cost.

The major strength of our study is that it is the first to assess cultural beliefs in Vietnamese women using a validated instrument that can contribute to the late detection of breast cancer and inform future studies in other areas in Vietnam and in other Asian countries. Nevertheless, there are several limitations to our study. The convenience sampling and cross-sectional design limits the generalizability of our study findings and do not allow for the identification of casual relationships among study variables. Second, the retrospective findings only provide a general overview of cultural beliefs and the factors associated with them. Further, our samples were recruited from one rural province, where residents have a low average income. Therefore, the study findings may not be generalized to other areas in Vietnam, especially to urban cities where residents have a higher income, and may not fully explain the cultural beliefs of all Vietnamese women. Future studies should employ larger samples with more complex sampling methods. Additional studies using larger samples and random sampling from other rural provinces may be helpful to identify the cultural beliefs of more Vietnamese women. Lastly, although we used a rigorous translation process including cognitive interviews, there is a possibility that we misinterpreted some concepts or did not include items that are specific to Vietnamese women since the original instrument was developed for minorities in the United States. More rigorous psychometric testing of the instrument is recommended for future studies.

## Conclusions

This study makes several contributions to the current cultural beliefs related BCS in Vietnamese rural women. We found the level of cultural beliefs about breast cancer in Vietnamese women were generally low, and also found that younger women and women with lower income had more erroneous cultural beliefs. Therefore, the findings support the need for health education that addresses the early detection of breast cancer including BSE and guidance on evidence-based, up-to-date, intervention measures that target women’s cultural beliefs, especially women aged younger than 40 years. Finally, it is vital for public health educators and administrators to understand that cultural beliefs about breast cancer in Vietnamese women differ by generation. Accordingly, a targeted approach to health education and BCS strategies is needed to support different generations of Vietnamese women to optimize breast cancer screening.

## Additional file

Additional file 1: Breast Cancer Cultural Beliefs Scale. (DOCX 16 kb)

## Data Availability

The datasets used and/or analyzed during the current study are not publicly available due to their contents containing personal information. However, redacted version may be made available from the corresponding author on reasonable request.

## Abbreviations

BCS: Breast Cancer Screening
BSE: Breast Self-Examination
