# Supplementary material for: Cultural beliefs about breast cancer in Vietnamese women
Source: BMC Womens Health. 2019 Jun 11;19:74. doi: 10.1186/s12905-019-0777-3 (PMC6558807; doi:10.1186/s12905-019-0777-3)
Supplement: Supplementary file 1 — Breast Cancer Cultural Beliefs Scale. (DOCX 16 kb) [file 12905_2019_777_MOESM1_ESM.docx]

**Tín ngưỡng văn hoá (Quan điểm) đối với bệnh ung thư vú_Huong**

Mỗi người có những quan điểm khác nhau về triệu chứng và cách điều trị ung thư vú. Chúng tôi muốn biết quan điểm của bạn. Theo bạn, những quan điểm sau là ĐÚNG hay SAI.

| Stt | Các phát biểu | Đúng | Sai |
| --- | --- | --- | --- |
| 1 | Nếu một khối u vú không bị đau, thì đó không phải là ung thư |  |  |
| 2 | Nếu một khối u vú không to lên, thì đó không phải là ung thư |  |  |
| 3 | Nếu một khối u vú bị chạm vào/ấn vào thường xuyên, khối u sẽ chuyển thành ung thư |  |  |
| 4 | Phụ nữ có vú to có khả năng bị ung thư vú cao hơn phụ nữ có vú nhỏ |  |  |
| 5 | Càng lo lắng về ung thư vú, bạn càng có nguy cơ bị ung thư vú |  |  |
| 6 | Nếu bạn chăm sóc bản thân mình tốt, bạn sẽ không bị ung thư vú |  |  |
| 7 | Có niềm tin vào Chúa (Trời phật) có thể bảo vệ bạn khỏi ung thư vú |  |  |
| 8 | Bạn chỉ cần chụp X quang vú nếu bạn phát hiện có bất thường ở vú của mình |  |  |
| 9 | Chụp X quang vú có thể gây ra ung thư vú |  |  |
| 10 | Nếu bạn chịu khó cầu nguyện thành tâm, có khả năng các khối u vú sẽ biến mất |  |  |
| 11 | Nếu bạn phẫu thuật cắt bỏ khối ung thư vú, nó sẽ phát triển nhanh hơn |  |  |
| 12 | Nếu gia đình bạn không có người bị ung thư vú, bạn không cần chụp X quang vú |  |  |
| 13 | Nếu bạn có một khối u vú, một phương thức chữa bệnh “gia truyền” có thể loại bỏ nó |  |  |
| 14 | Nếu một người phụ nữ có đủ lòng tin vào Chúa (Trời phật), cô ấy sẽ không cần chữa trị ung thư vú |  |  |
| 15 | Nếu một người phụ nữ nghèo, cô ấy sẽ không chữa khỏi được bệnh ung thư, vì cô ấy sẽ không nhận được sự điều trị tốt nhất |  |  |
| 16 | Nếu ung thư vú được điều trị đúng cách, bệnh có thể được chữa khỏi |  |  |
| 17 | Điều trị bệnh ung thư vú là không thực sự giải quyết được vấn đề, bởi vì nếu bạn bị ung thư, không sớm thì muộn bạn cũng sẽ chết vì nó |  |  |
